# A three-gene cluster in *Trichoderma reesei* reveals a potential role of *dmm2* in DNA repair and cellulase production

**DOI:** 10.1186/s13068-022-02132-y

**Published:** 2022-03-29

**Authors:** Wanchuan Cai, Yumeng Chen, Lei Zhang, Xu Fang, Wei Wang

**Affiliations:** 1 State Key Lab of Bioreactor Engineering, New World Institute of Biotechnology, East China University of Science and Technology, Shanghai, 200237 China; 2 State Key Laboratory of Microbial Technology, School of Life Sciences, Shandong University, Jinan, China; 3 State Key Lab of Bioreactor Engineering, New World Institute of Biotechnology, East China University of Science and Technology, 130 Meilong Road, P.O. Box 311, Shanghai, 200237 China

**Keywords:** *Trichoderma reesei*, DNA methylation modulator-2, *tku70*, *Hem8*, *dmm2*, Ferrochelatase, Cellulase

## Abstract

**Background:**

The ascomycete *Trichoderma reesei* is one of the most efficient industrial producers of cellulase. Gene targeting by homologous recombination is a key technique for improving strains and constructing mutants. In *T. reesei*, *tku70* (homologous to human KU70) was deleted to block non-homologous end-joining, which led to 95% of transformants exhibiting homologous recombination.

**Results:**

Two genes located in close proximity to *tku70* were identified: the ferrochelatase gene *hem8* (*tre78582*, homologous to *Aspergillus niger hemH* and *Cryptococcus neoformans HEM15*) and a putative DNA methylation modulator-2 gene *dmm2* (*tre108087*, homologous to *Neurospora crassa dmm-2*). Genome-wide surveys of 324 sequenced fungal genomes revealed that the homologues of the three genes of interest are encoded in tandem in most *Sordariomycetes*. The expression of this three-gene cluster is regulated by blue light. The roles of these three genes were analyzed via deletion and complementation tests. The gene *hem8* was originally described as a novel and highly distinct auxotrophic marker in *T. reesei* and we found that the product protein, HEM8, catalyzes the final step in heme biosynthesis from highly photoreactive porphyrins. The lethal phenotype of the *hem8* deletion could be overcome by hematin supplementation. We also studied the functions of *tku70* and *dmm2* in DNA repair using mutagen sensitivity experiments. We found that the Δ*tku70* strain showed increased sensitivity to bleomycin, which induces DNA double-strand breaks, and that the Δ*dmm2* strain was sensitive to bleomycin, camptothecin (an inhibitor of type I topoisomerases), and hydroxyurea (a deoxynucleotide synthesis inhibitor). The double-mutant Δ*tku70*&*dmm2* showed higher sensitivity to hydroxyurea, camptothecin, and bleomycin than either of the single mutants. Knockout of *dmm2* significantly increased cellulase production.

**Conclusions:**

Our data show, for the first time, that ferrochelatase encoded by *hem8* catalyzes the final step in heme biosynthesis from highly photoreactive porphyrins and that *dmm2* encodes a putative DNA methylation modulator-2 protein related to DNA repair and cellulase expression in *T. reesei*. Our data provide important insights into the roles of this three-gene cluster in *T. reesei* and other *Sordariomycetes* and show that the DNA methylation modulator DMM2 affects cellulase gene expression in *T. reesei*.

**Supplementary Information:**

The online version contains supplementary material available at 10.1186/s13068-022-02132-y.

## Background

The ascomycete *Trichoderma reesei* is one of the most efficient industrial producer of cellulolytic enzymes [1]. Owing to the increased need for enhanced productivity of cellulases, researchers have focused on improving this strain [1–3]. Gene targeting by homologous recombination (HR) is a key technique used to improve strains, and researchers have attempted to achieve high HR rates to construct mutants [4, 5].

In eukaryotes, two main recombination pathways have been identified: the HR pathway and non-homologous end-joining (NHEJ) pathway [6], which differ based on whether the DNA double-strand breaks (DSBs) repair is dependent on DNA sequence homology or not. KU70 is one of the DNA repair proteins required for the NHEJ pathway [7]. Mutations in these proteins lead to mild sensitivity to DNA-damaging agents such as UV irradiation, methyl methanesulfonate (MMS), ethyl methanesulfonate (EMS), and phleomycin/bleomycin [8–10]. Earlier studies have also shown that the homologous integration frequency via the HR pathway can be improved in mycelial fungi by interrupting the NHEJ pathway [11]. In *T. reesei*, *tku70* (homologous to human *KU70*) has been deleted to block NHEJ repair, resulting in integration at the homologous site (HR pathway) in over 95% of the transformants [4, 5].

Heme biosynthesis, a multistep biosynthetic pathway, is highly conserved and tightly regulated throughout evolution. Ferrochelatase participates in the final step of heme biosynthesis and catalyzes the insertion of ferrous iron into protoporphyrin IX [12]. Light can kill or mutate organisms by inducing the generation of highly phototoxic porphyrin molecules during heme biosynthesis [12]. In *Cryptococcus neoformans*, *Neurospora crassa*, and *Phycomyces blakesleeanus*, which are representative species from three diverse phyla of the fungal kingdom, ferrochelatase is produced in response to light as a means of reducing exposure to photosensitization via modulation of porphyrins [12, 13]. Deletion of ferrochelatase *hemH* in *Aspergillus niger* is conditionally lethal, and despite supplementation with hemin, the Δ*hemH* strain still exhibits extremely impaired growth [14]. In *C. neoformans*, ferrochelatase-deletion mutants Δ*HEM15* are unable to generate haploid strains, even when the medium is supplemented with exogenous hemin [13]. The mechanisms and regulation of heme biosynthesis in *T. reesei* have not been elucidated yet.

Many organisms use DNA methylation to silence transcriptional genes. In *N. crassa*, DNA Methylation Modulators (DMMs) prevent aberrant spreading of DNA methylation from normally methylated A:T-rich DNA to nearby genes [15, 16]. DMMs rely on an associated protein, DMM-2, with a fungal-specific Zn(II)_2_Cys_6_ DNA-binding motif, required for localization and proper function [15, 16]. Mutations in DMM-2 resulted in DNA hypermethylation defects in *N. crassa* [15, 16]. However, the mechanisms that restrict methylation to appropriate regions are largely unknown in *T. reesei*.

In our previous study, we attempted to delete the DNA repair protein encoded by the *tku70* gene to block NHEJ repair and improve homologous integration in the hypersecreting mutant *T. reesei* RUT-C30 (ATCC 56765). However, we failed to knock out the complete coding region of *tku70*, which implied that some essential genes are located around *tku70* and that the complete deletion of this region will affect their expression. In this study, we identified a three-gene cluster around *tku70*, which includes the ferrochelatase gene *hem8* (*tre78582*), *tku70*, and a putative DNA Methylation Modulator-2 gene *dmm2* (*tre108087*). Genome-wide surveys of 324 sequenced fungal genomes revealed that the homologues of the three genes of interest are encoded in tandem in most *Sordariomycetes*. Our findings demonstrate, for the first time, that ferrochelatase encoded by *hem8* catalyzes the final step in heme biosynthesis and that *dmm2* is related to DNA repair and cellulase expression in *T. reesei*. These results suggest a potential role for DMM2 and DNA methylation in DNA repair and cellulase production in *T. reesei*.

## Results

### Identification of a three-gene cluster and phylogenetic analysis of *tre78582* and *tre108087*

**Fig. 1:**
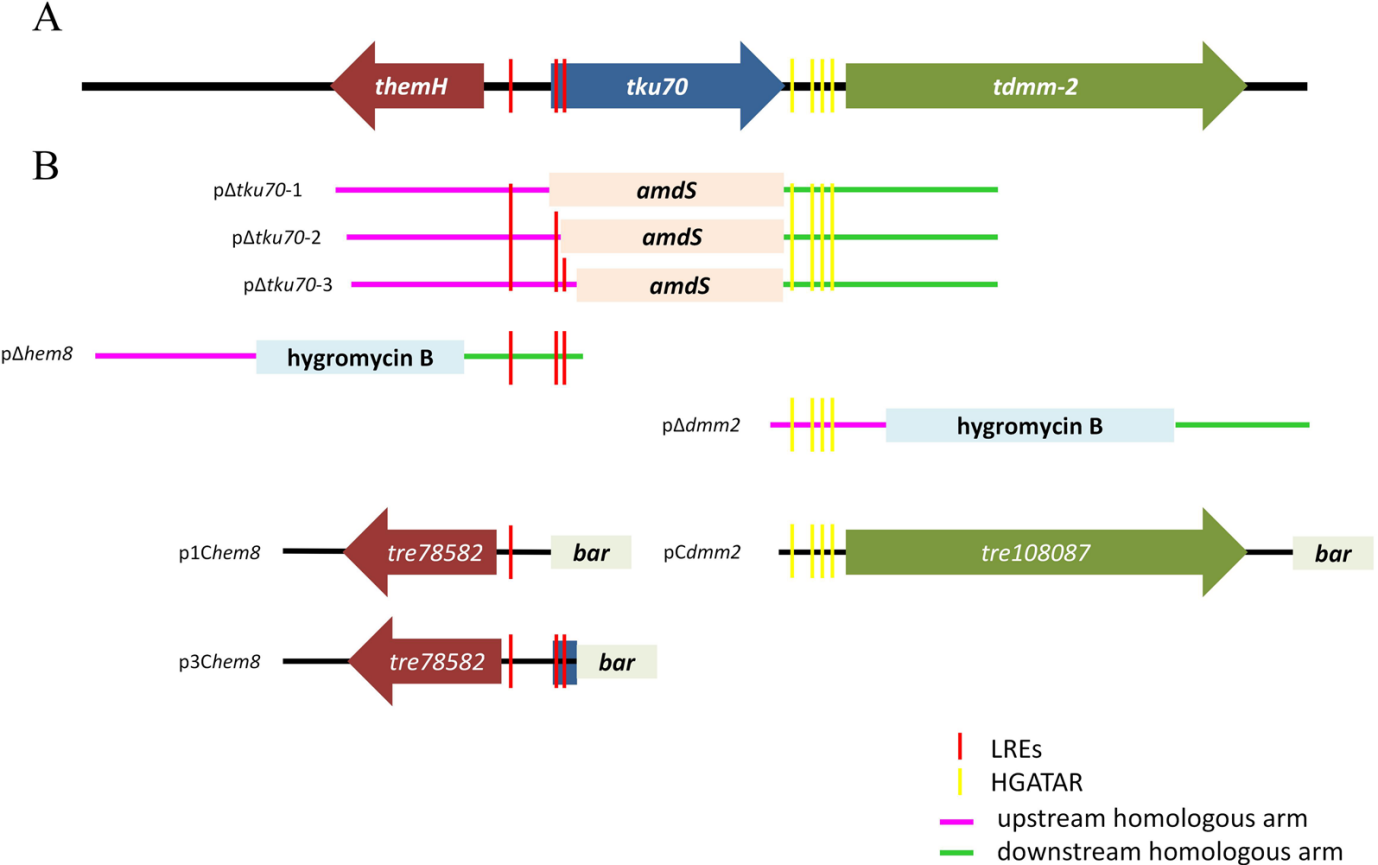
Schematic presentation of the constructs used in this study. **A** Genomic organization of *hem8*, *tku70*, and *dmm2*. **B** Design of deletion and complementation cassettes. Orientation of the genes is indicated by arrows. Colored vertical bars indicate positions of specific motifs, including light-response elements (LREs) and the HGATAR (H = C, T, A; R = A, G) consensus sequence. Colored horizontal bars indicate homologous arms with different lengths

A deletion plasmid pΔ*tku70*-1 was constructed with 2.0-kb upstream and downstream of the coding region of *tku70* used for the 5′- and 3′-flanking regions, respectively, to knock out the complete *tku70* coding region (Fig. 1B). After transformation, 24 transformants were selected; however, it was not possible to identify positive *tku70* deletion mutants in the PCR screen (data not shown). These results imply that some essential genes are located around *tku70* and that deleting the complete *tku70* coding region affects their expression, causing the mutants to fail to grow (Fig. 1).

BLASTP analysis showed that *tre78582* (scaffold_11:161209-162603; XP 006966087.1), the upstream gene of *tku70*, encodes a sequence of 421 amino acids that belongs to the chelatase class II family (ferrochelatase; pfam00762; *E*-value, 1.92e^−126^). This sequence shares 78% identity with HemH (NCBI Reference Sequence: XP_001396807.1), a ferrochelatase identified in *A*. *niger*. Additionally, *tre108087* (scaffold_11:165570-169162; XP 006965972.1), the hypothetical downstream gene of *tku70*, encodes a putative DNA methylation modulator-2 with a peptide of 1152 amino acids and has a GAL4-like Zn(II)_2_Cys_6_ binuclear cluster DNA-binding domain (pfam00172; *E*-value, 5.08e^−11^). The closest match to the deduced amino acid sequence available in the databases was a hypothetical protein (XP_013954510.1) in *T. virens* Gv29-8, with 64% identity.

**Fig. 2:**
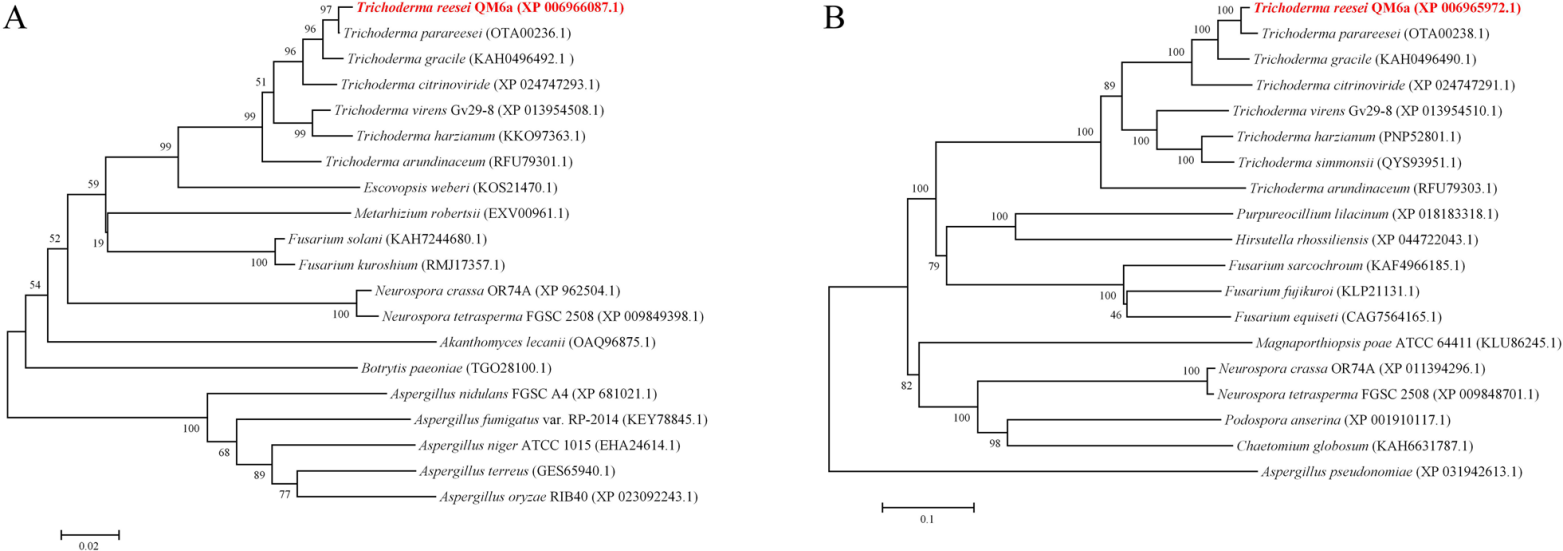
Phylogenetic analysis of HEM8 (**A**) and DMM2 (**B**). Alignment of sequences was performed with ClustalW. Phylogenetic and molecular evolutionary analyses were conducted using MEGA6. The numbers below the nodes indicate the bootstrap values. The scale bar reflects the evolutionary distance

Phylogenetic analysis of Trire2:78582 and Trire2:108087 protein sequences (Fig. 2) produced a tree whose branching was consistent with the established phylogenetic relationships between the various taxa, indicating orthology of the identified protein sequences. Thus, we considered Trire2:78582 as the HemH ortholog in *T. reesei* and designated the protein as HEM8 (gene name: *hem8*) and Trire2:108087 as the DMM-2 [15, 16] ortholog in *T. reesei* and designated the protein as DMM2 (gene name: *dmm2*).

The three genes—*tku70*, *hem8*, and *dmm2*—showed a very small physical distance between them (Fig. 1). Genes *hem8* and *tku70* were 496 bp apart, and *tku70* and *dmm2* were 393 bp apart. The orientations of *tku70* and *hem8* are inverted, and a bidirectional promoter (496 bp) was identified between these two genes. Moreover, *dmm2* was located on the same DNA strand as *tku70*.

Genome-wide surveys (Additional file 1) of 324 sequenced fungal genomes from *Ascomycota*, *Basidiomycota*, *Blastocladiomycota*, *Chytridiomycota*, *Glomeromycota*, *Neocallimastigomycota*, and *Zygomycota* revealed some interesting observations. First, the homologues of our three genes of interest are encoded in tandem in most *Sordariomycetes*. Additionally, in 75% (136/180) of ascomycetous fungal genomes, orthologs of *hem8* and *tku70* are very close (not more than 1000 bp apart) and encoded in two different DNA strands. The two adjacent genes may share a common promoter, since the intergenic region of the two genes is very short. We also observed that there is only one ortholog of *hem8* or *tku70* in most ascomycetous fungi, including *T. reesei*. Moreover, several fungi from ascomycete and basidiomycete phyla have two *hem8* orthologs (such as *Aspergillus zonatus* and *Penicillium raistrickii*) or two *tku70* orthologs (such as *Candida arabinofermentans* and *Plicaturopsis crispa*). Another interesting observation was the absence of the *tku70* ortholog in some *Saccharomycetales* species (such as *Pichia membranifaciens* and *Hyphopichia burtoni*), which is consistent with the predominance of HR over NHEJ in these species [17]. Furthermore, only one conserved domain, the Zn(II)_2_Cys_6_ binuclear cluster DNA-binding domain, was found located in the middle of the predicted protein sequence of DMM2. We could observe that this non-N/C-terminal Zn(II)_2_Cys_6_ protein is widely distributed in ascomycetous fungal genomes (present in 87 of 180 genomes). Only two species of *Basidiomycota*, *Amanita thiersii* and *Gymnopus luxurians*, have orthologs of DMM2.

### *hem8 *and* tku70* are transcriptionally regulated by light

**Fig. 3:**
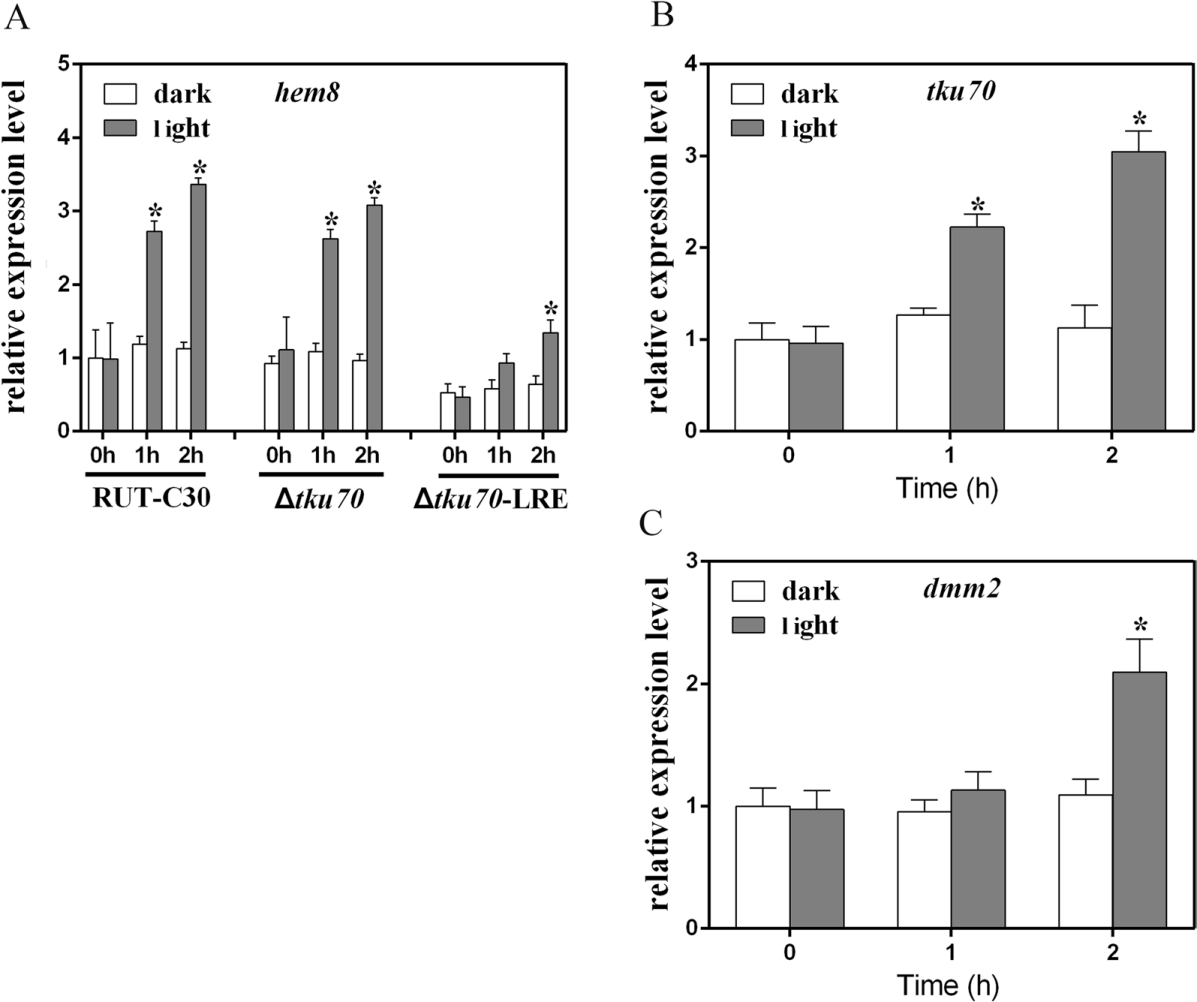
Expression levels in the absence and presence of blue light. Quantitative PCR(qPCR) was used to detect the transcriptional levels of three genes. The relative transcript levels of *hem8* (**A**), *tku70* (**B**), and *dmm2* (**C**) at 0, 1, and 2 h for blue light cultivation were analyzed. The mRNA level of the reference gene *rpl6e* was set as an endogenous control in all samples. Error bars indicate the means ± SEMs (*n* = 3 samples) from the same experiment. Asterisks indicate significant differences from the corresponding dark cultivation (*P < 0.05) based on one-way analysis of variance

Earlier studies have shown that some *hemH* genes in fungi are under the control of a light-regulated promoter to reduce photo-oxidative damage via light-mediated action on porphyrins [13]. *Trichoderma reesei hem8* and *tku70* share a 496-bp upstream noncoding region, which is thought to be a bidirectional promoter (Fig. 1). Therefore, we hypothesized that *tku70* may also be regulated by light. To evaluate this assumption, we compared transcript levels in the presence or absence of blue light using RT-qPCR. As shown in Fig. 3A, B, *hem8* and *tku70* were properly photo-induced by 2- to 3-fold, when compared with the mRNA levels in darkness, indicating that their expression was strongly affected by blue light. The *hem8* transcript level was increased by 2.8-fold within the first hour under illumination, showing a minor short-term response to light and a slight increase after constant long-term illumination conditions (Fig. 3B). In contrast, *dmm2* was induced twofold after 2 h (Fig. 3C). Hence, these genes are regulated in response to light, with variable time required for induction in each case.

The promoter regions of the three genes were analyzed for specific motifs, including the light-response element (LRE), a DNA motif found in the promoters of genes regulated by light [18–21], and the fungal GATA factor DNA-binding HGATAR (H = C, T, A; R = A, G) consensus sequences [22]. As presented in Fig. 1A, one LRE motif was found in the bidirectional promoter region. Additionally, four HGATAR-binding sites were found in the *dmm2* promoter region. These data correlate with the effects of light on *tku70*, *hem8*, and *dmm2* transcripts, as detected by RT-qPCR. Furthermore, we found two LRE motifs in the 5′-coding region of *tku70* (Fig. 1A). In eukaryotic cells, overlapping genes are not common and we set out to determine whether the two LRE motifs overlapping with *tku70* are also responsible for light-regulated transcription.

Two other *tku70* deletion plasmids, pΔ*tku70*-2 and pΔ*tku70*-3, were constructed with different 5′-flanking regions than the one in pΔ*tku70*-1 (Fig. 1B). In pΔ*tku70*-1, the 2.0-kb upstream noncoding region of *tku70* was used as the 5′-flanking region to knock out the complete *tku70* coding region, including the two LRE motifs (Fig. 1B). In pΔ*tku70*-2 and pΔ*tku70*-3, a small portion of 5′-*tku70*, which included only one or two of the LRE motifs, was used to knock out partial *tku70* coding regions reserving one or two LRE motifs, respectively (Fig. 1B). After transformation, 24 transformants were screened for *tku70* deletion by PCR (data not shown). Homologous integration rates were markedly different between pΔ*tku70*-1 (no *tku70* deletion mutant from 24 transformants), pΔ*tku70*-2 (five *tku70* deletion mutants from 24 transformants, which were named Δ*tku70-*LRE strains with two LRE motifs left in the 5′-flanking region of *hem8*), and pΔ*tku70*-3 (12 *tku70* deletion mutants from 24 transformants, which were named Δ*tku70* strains with all three LRE motifs left in the 5′-flanking region of *hem8*). We compared the transcript levels of *hem8* in Δ*tku70* and Δ*tku70-*LRE using RT-qPCR. As shown in Fig. 3A, photoinduction of *hem8* was partially compromised in Δ*tku70-*LRE compared to that in Δ*tku70,* and the parental strain RUT-C30 for one LRE motif was deleted in the Δ*tku70-*LRE strain. These results further support the notion that LRE motifs overlapping with *tku70* are crucial for the full transcription of ferrochelatase *hem8*.

### *hem8* encodes a putative ferrochelatase, an essential protein in *T. reesei*

Initial attempts to delete *hem8* by transforming pΔ*hem8* into the Rut-C30 strain failed. Twenty-four transformants were screened for deletion of *hem8* and no mutant was identified, suggesting that ferrochelatase is essential for *T. reesei.* We were also unable to isolate *hem8* deletion mutants in *T. reesei* haploid strains through conventional gene replacement experiments, using HR, even when the medium was supplemented with exogenous hematin. Earlier studies have reported that the use of the KU70 ortholog deletion strain results in a high frequency of heterokaryon formation in primary transformants when an essential gene is disrupted [23]. We speculated that it would be possible to select homokaryons from viable heterokaryons. Therefore, we first attempted to obtain the *hem8* deletion strain based on the *tku70* deletion mutant Δ*tku70*.

After transforming the Δ*tku70* strains, the colonies (heterokaryons) displaying slightly red autofluorescence under 365-nm light were selected for further spore production (Additional file 2: Fig. S1). Deletion of *hem8* is conditionally lethal, and supplementation with hematin yields brownish-pink colonies, indicating porphyrin accumulation in the cells [24]. However, our initial attempts to isolate Δ*hem8* homokaryons were unsuccessful. After one round of single conidiospore isolation, no colonies germinated in Mandels medium supplemented with 50 mg/L hygromycin and 100 mg/L hematin. Therefore, we questioned whether *T. reesei* spores were not capable of hematin uptake for germination.

**Fig. 4:**
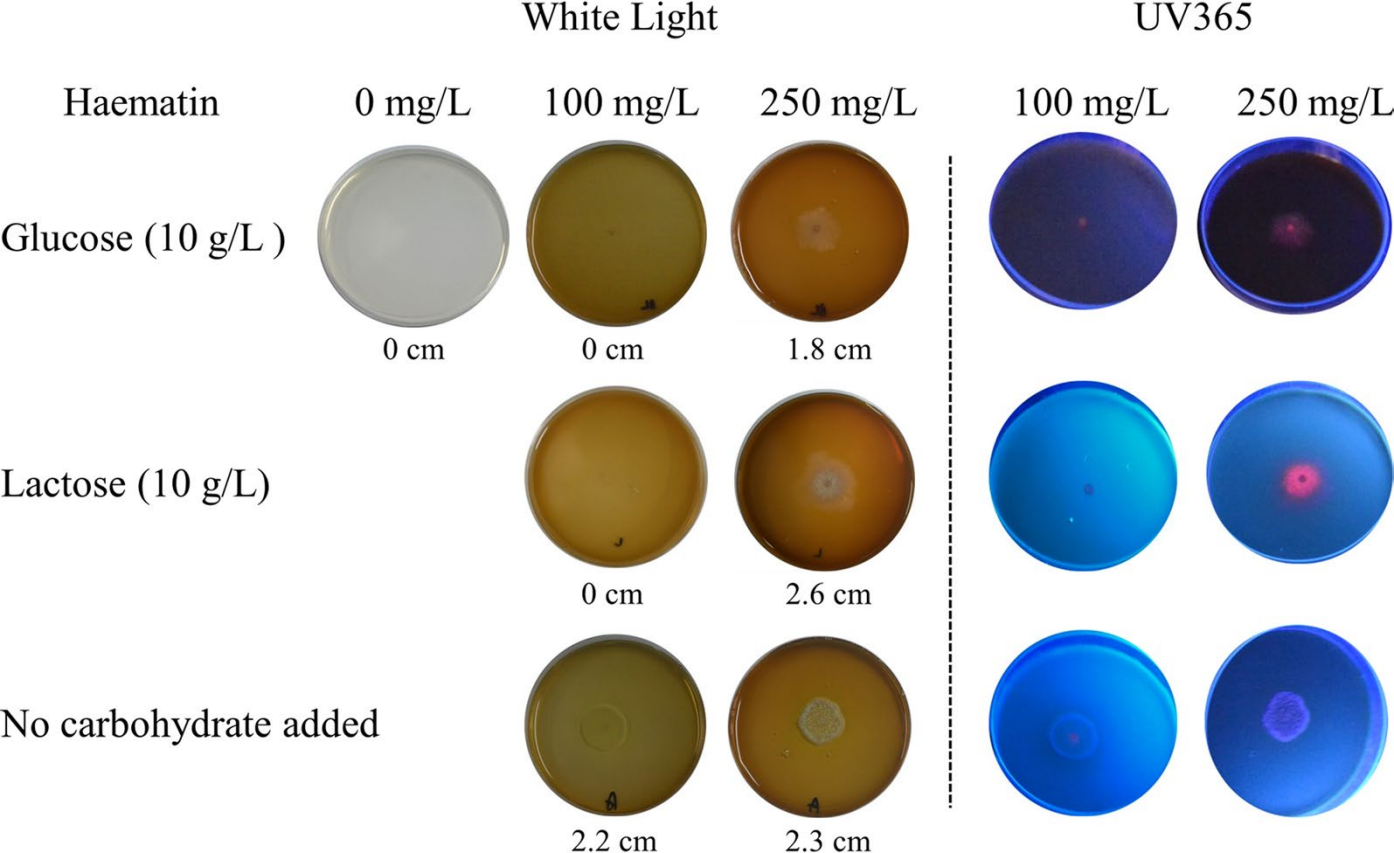
Phenotypic comparison and fluorescence detection of *hem8* deletion strains. Conidia (5 × 10^3^) were spotted, and plates were incubated at 28 °C for 3–5 days. Strains were inoculated onto malt extract agar plates containing 0, 100, and 250 mg/L hematin. These malt extract agar plates were also supplemented with different carbon sources (glucose, lactose, or no carbohydrate added). Red autofluorescence was detected under 365 nm UV light. Colony diameters are indicated below the plates

Notably, we changed Mandels medium to malt extract agar and, under this condition, we were able to successfully isolate homokaryons (Additional file 2: Fig. S1). As the greatest difference between Mandels medium and malt extract agar is the carbon source, we decided to examine the additive carbon source and the concentration of hematin, to determine the optimal culture conditions for the deletion mutants. Conidia (5 × 10^3^) of Δ*hem8* strains were transferred to different malt extract agar supplemented with 0, 100, or 250 mg/L of hematin and 0 or 10 g/L of glucose or lactose incubated at 28 °C for 3 days in the dark. We observed that the higher the concentration of hematin in the medium, the faster the strains grew. No growth was observed when spores were inoculated on medium without hematin (Fig. 4). Moreover, no obvious growth was observed when spores were inoculated on medium containing 100 mg/L of hematin and 10 g/L of carbohydrates (Fig. 4). Red fluorescence under 365-nm light indicated dramatic accumulation of porphyrin (Fig. 4), revealing the absence of growth due to upregulation of porphyrin, which would be expected to result in increased damage, even under dark conditions. However, when the concentration of hematin was increased to 250 mg/L, Δ*hem8* could grow in the presence of 10 g/L of carbohydrates (Fig. 4). These results indicate that the addition of glucose or lactose induce porphyrin synthesis, whereas the addition of hematin inhibits porphyrin synthesis.

Complementation of *hem8* was done to analyze the effect of *hem8* on heme biosynthesis and test the possibility of its application as a selectable marker. The cassette p3C*hem8* (Fig. 1B) effectively complemented the growth defect in the absence of hematin (Additional file 2: Fig. S2). However, no obvious growth was observed when the Δ*hem8* strain was transformed with the p1C*hem8* cassette (Fig. 1B) and grown on plates without hematin, except for a few colonies exhibiting slow growth (Additional file 2: Fig. S2). After 5 days of incubation, these slow-growing colonies accumulated large amounts of porphyrin (Additional file 2: Fig. S2). We reasoned that this difference results from the fact that the p1C*hem8* cassette has only one LRE motif (from the 496-bp bidirectional promoter between *hem8* and *tku70*), whereas p3C*hem8* complements the function of *hem8* with three LRE motifs (including two motifs overlapped with the ORF of *tku70*). The presence of only one LRE motif in the promoter may not be sufficient for complementation of *hem8* expression similar to the parental strain, as the resulting colonies grew slowly and emitted high fluorescence (Additional file 2: Fig. S2), indicative of a dramatic accumulation of porphyrin. Thus, by deletion of *tku70* and complementation of the Δ*hem8* mutant, we demonstrate that the *hem8* gene encodes a functional ferrochelatase enzyme, and that the two LRE motifs at the coding region of *tku70* are essential for ferrochelatase *hem8* transcription. These data suggest that *hem8* is a novel and highly distinct auxotrophic marker in *T. reesei*. The partial complementation strain Δ*hem8*::p1C*hem8* might be a good tool for porphyrin production.

Combined with previous data describing the increased sensitivity to UV irradiation of Δ*tku70* strains [4, 23], our data suggest that light sensing by *tku70 and hem8* is involved in protection against damage caused by UV light. Thus, we decided to investigate whether light-inducible *dmm2* is also responsible for DNA repair.

### *dmm2* is involved in DNA repair

KU70 is a key component of the NHEJ for DSB repair. Previous data show that deletion of the *tku70* gene increases sensitivity to UV irradiation [4]. To systematically study the role of *tku70* and *dmm2* in DNA repair, we constructed three mutants: Δ*tku70*, Δ*dmm2*, and the double-deletion strain Δ*tku70*&*dmm2*. Deletion of *dmm2* (Fig. 2B) did not affect the growth on agar plates or biomass production in shake-flask cultures.

**Fig. 5:**
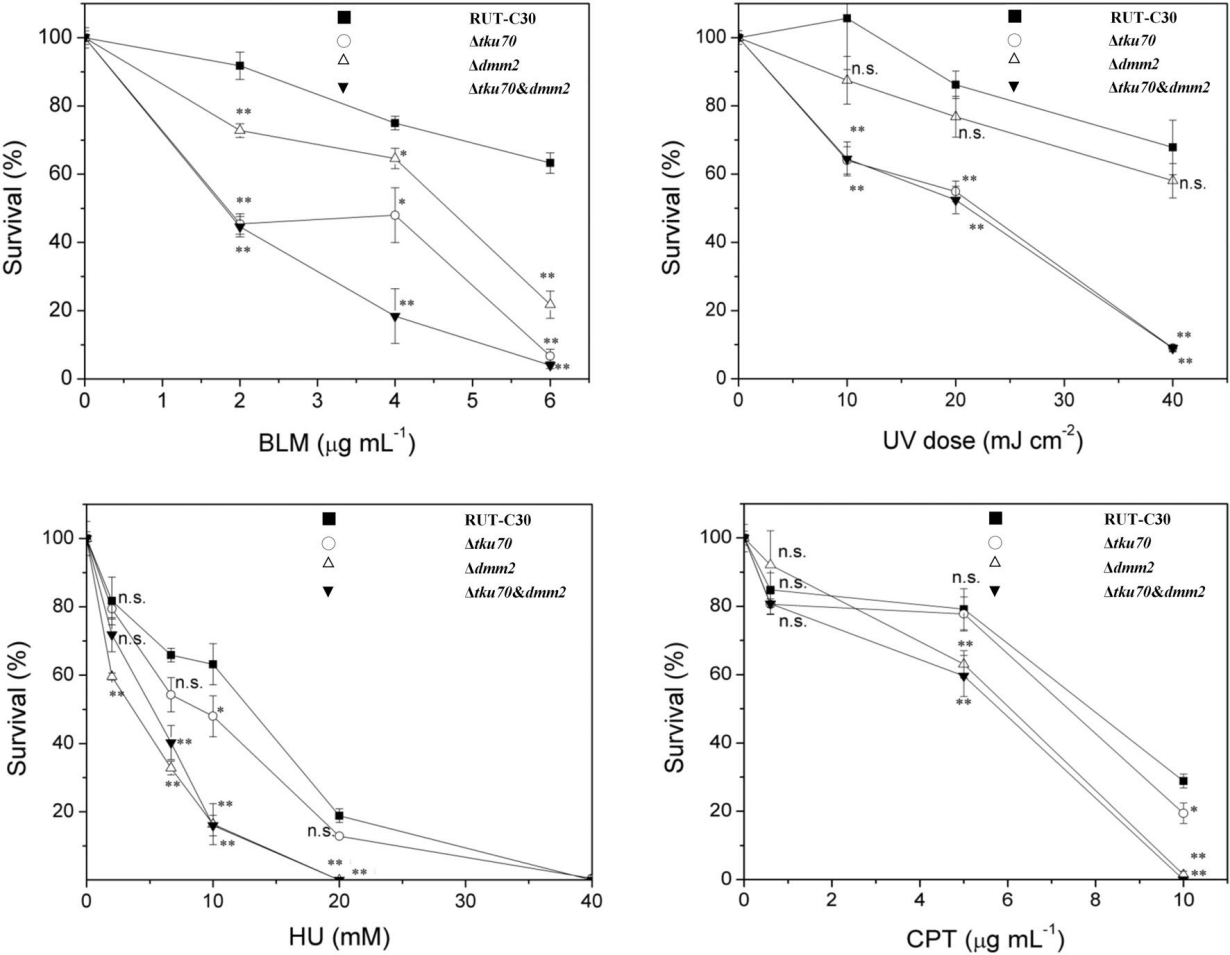
Sensitivities and epistasis analysis of Δ*tku70* and Δ*dmm2*. A conidial suspension was irradiated with UV at the indicated dose or mixed with malt extract agar medium containing the colony restrictor Triton X-100 and BLM, HU, or CPT at the indicated concentration. Colonies were counted after incubation at 28 °C for 2–3 days. Error bars indicate means ± SEMs (*n* = 3 samples) from the same experiment. Asterisks indicate significant differences from the parental strain RUT-C30 (*P < 0.05; **P < 0.01; *ns* not significant) based on one-way analysis of variance

Next, we tested the sensitivity of Δ*tku70*, Δ*dmm2*, and the parental strain RUT-C30 to different mutagens such as hydroxyurea (HU) [8], MMS, EMS, bleomycin (BLM), camptothecin (CPT) [8], mitoxantrone (MIT) [8], and UV (Fig. 5 and Additional file 2: Fig. S3). Epistatic relationships between *dmm2* and *tku70* were examined using the double-mutant Δ*tku70*&*dmm2.* The *T. reesei* Δ*tku70* strain showed increased sensitivity to BLM, when compared to the parental RUT-C30 strain (Fig. 5). Moreover, the *T. reesei* Δ*tku70* strain showed a slightly increased sensitivity to CPT and HU, when compared to the parental strain (Fig. 5, Additional file 2: Fig. S3). We reconfirmed that the *T. reesei* Δ*tku70* strain showed increased sensitivity to UV (Fig. 5) and no obvious increased sensitivity to MMS (Fig. 5, Additional file 2: Fig. S3). Finally, we found that the *T. reesei* Δ*tku70* strain showed no obvious increase in sensitivity to EMS and MIT (Additional file 2: Fig. S3). Interestingly, while the *T. reesei* Δ*dmm2* strain showed no increased sensitivity to MMS, EMS, and MIT, we could observe increased sensitivity to BLM (Fig. 5). We also found that the *T. reesei* Δ*dmm2* strain showed sensitivity to HU and CPT, while the Δ*tku70* strain showed slightly increased sensitivity (Fig. 5). In contrast, the *T. reesei* Δ*dmm2* strain showed a mild sensitivity to UV compared with the parental strain, while the Δ*tku70* strain showed sensitivity (Fig. 5).

Double mutants were constructed by combining Δ*tku70* and Δ*dmm2* with DNA repair mutants representing various epistatic groups and determining the sensitivity of each strain to UV, HU, CPT, and BLM. The *tku70* mutation was epistatic to the *dmm2* mutation in terms of sensitivity to UV radiation (Fig. 5). In contrast, the *dmm2* mutation was epistatic to the *tku70* mutation in terms of sensitivity to HU and CPT (Fig. 5). The double-mutant was more sensitive to the DSB inducer BLM than the parental single mutant (Fig. 5). The results showed that the epistatic effect of BLM sensitivity was not significant. These observations suggest that *tku70* and *dmm2* are involved in different DSB repair pathways or different steps in DSB repair.

**Fig. 6:**
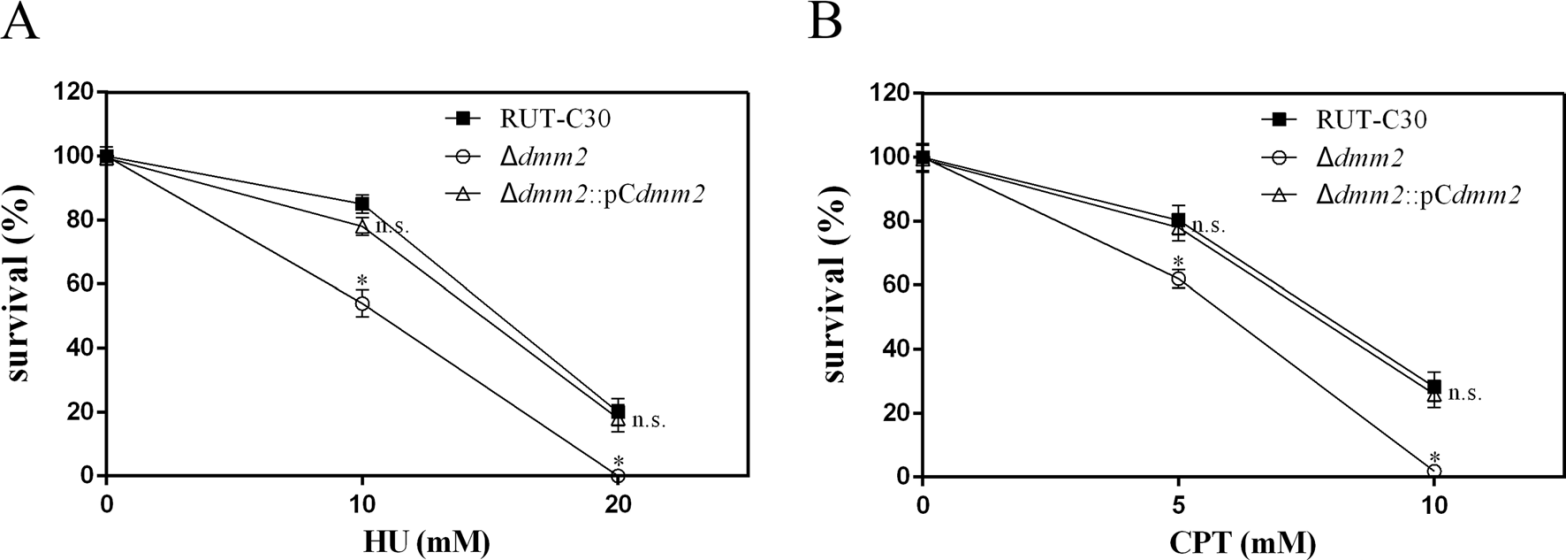
HU (**A**) and CPT (**B**) sensitivity of Δ*dmm2* complementation strains. Error bars indicate means ± SEMs (*n* = 3 samples) from the same experiment. Asterisks indicate significant differences from RUT-C30 (*0.01 < *P* < 0.05; **P < 0.01; *ns* not significant) based on one-way analysis of variance

To analyze the effects of *dmm2* on the sensitivity to HU and CPT, we tested the complementation of deletion strain Δ*dmm2* by *dmm2.* In fact, our results showed that pC*dmm2* (Fig. 1B) was able to rescue the sensitivity to HU and CPT of the Δ*dmm2* strain (Fig. 6). Thus, by deletion and complementation tests, we demonstrated that *dmm2* plays an important role in DNA repair in ascomycetous fungi.

Overall, our findings suggest that *dmm2* encodes a putative DNA methylation modulator-2 gene that has not been reported previously. *dmm2* may positively affect the expression of genes involved in DNA biosynthesis and topoisomerase type I activation to repair DSBs, as supported by the observation that the *dmm2* mutant displayed sensitivity to the deoxynucleotide synthesis inhibitor HU and topoisomerase type I inhibitor CPT.

### *dmm2* affects cellulase expression

**Fig. 7:**
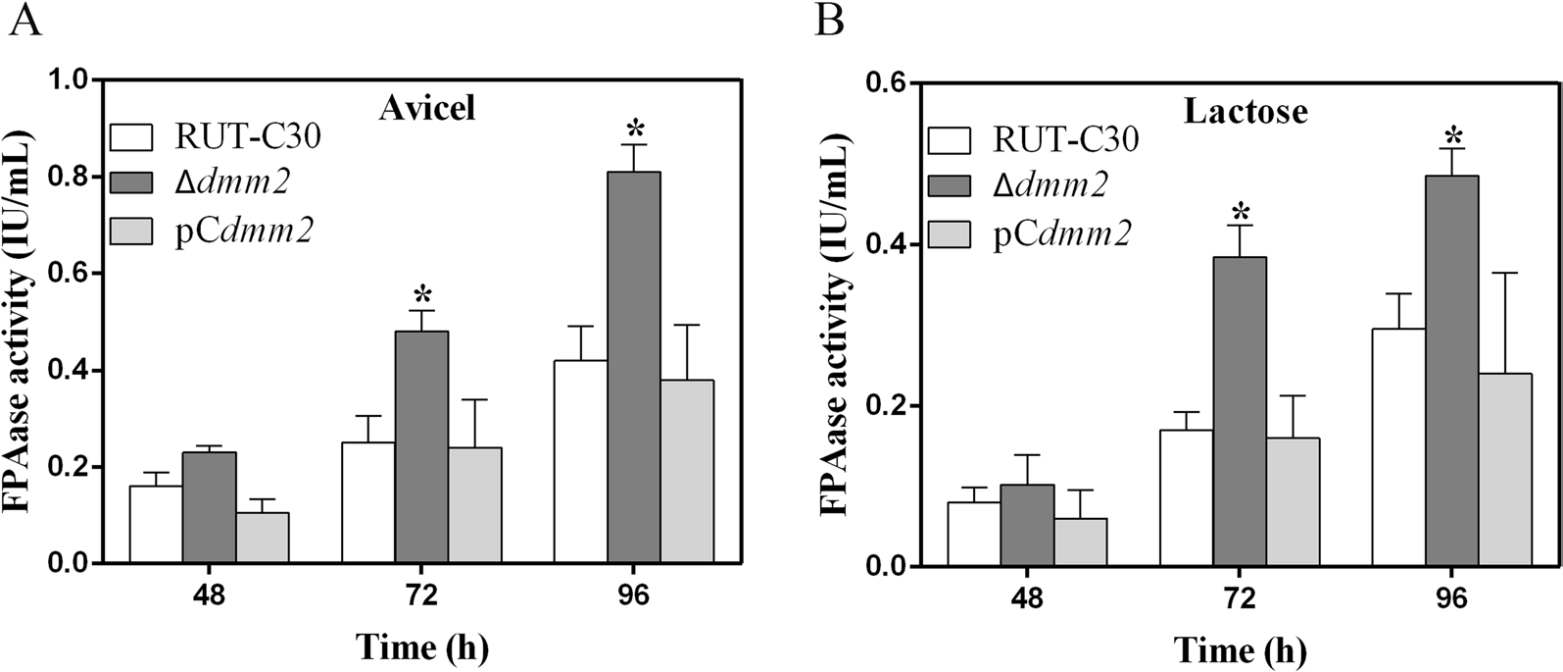
FPAase activities in RUT-C30, Δ*dmm2*, and pC*dmm2* incubated in shake-flask cultures with Avicel (**A**) and lactose (**B**). The activity was determined in pH 5.0 at 50 °C. All error bars indicate mean ± SEM (*n* = 3 samples) from the same experiment. Asterisks indicate significant differences from the parental strain RUT-C30 (*P < 0.05) based on one-way analysis of variance

**Fig. 8:**
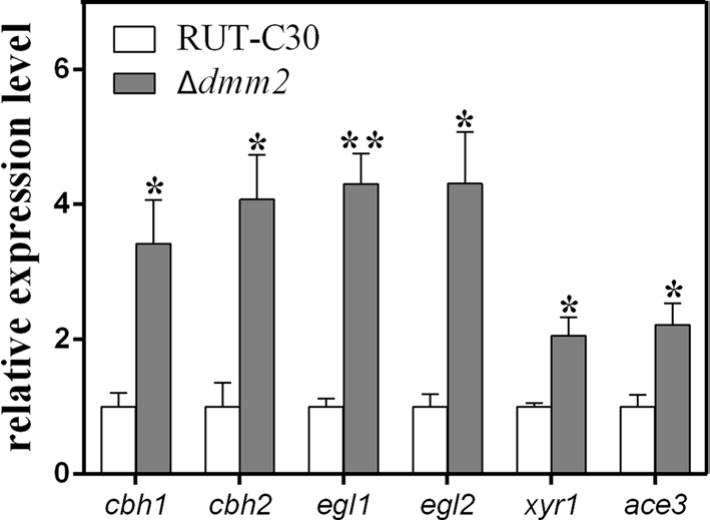
Expression levels of major cellulase genes and cellulase activator genes. Quantitative PCR (qPCR) was used to detect the transcriptional levels of four major cellulase genes (**A**)—*cbh1*, *cbh2*, *egl1*, and *egl2*—and two crucial cellulase activator genes (**B**)—*xyr1* and *ace3*. Conidia of *T. reesei* were inoculated into Mandels medium with Avicel as the sole carbon source. Sampling was done at 48 h. The data are normalized to expression of the parental strain RUT-C30 for each tested gene, with the *sar1* gene used as an endogenous control in all samples. Values are represented means ± SD of the results from three independent experiments. Asterisks indicate significant differences compared to parental strain (*P < 0.05; **P < 0.01, Student’s *t* test)

The deletion of *dmm2* had no effect on biomass production in shake-flask cultures with lactose (Additional file 2: Fig. S4), with the exception of a significant improvement (*P* < 0.05) in cellulase production when compared to that of the *T. reesei* parental strain RUT-C30 (Fig. 7). The *dmm2* deletion mutant, Δ*dmm2*, displayed significantly increased cellulase activity (150–200%), when compared to the parental strain RUT-C30, in the presence of microcrystalline cellulose (Avicel) or lactose (Fig. 5). The enzyme activity in the complementation strain pC*dmm2* decreased to the same level as that in the control RUT-C30 (Fig. 7). To investigate the effect of *dmm2* deletion over cellulase transcription, we used real-time fluorescent quantitative PCR (RT-qPCR) to detect the expression of major cellulase genes in the Δ*dmm2* strain. As shown in Fig. 8, knockout of *dmm2* significantly increased the expression of four major cellulase genes (*cbh1*, *cbh2*, *egl1*, and *egl2*). These results are consistent with the increased cellulase activity in the Δ*dmm2* strain (Fig. 7). XYR1 and ACE3 are considered crucial cellulase activators and both *xyr1* and *ace3* genes were significantly upregulated by two times (Fig. 8). In conclusion, the observed increase in cellulase production in the Δ*dmm2* strain may be due to the elevated expression of both cellulase and cellulase activator genes. These findings indicated that the DNA methylation modulator *dmm2* plays an inhibitory role in cellulase production.

**Fig. 9:**
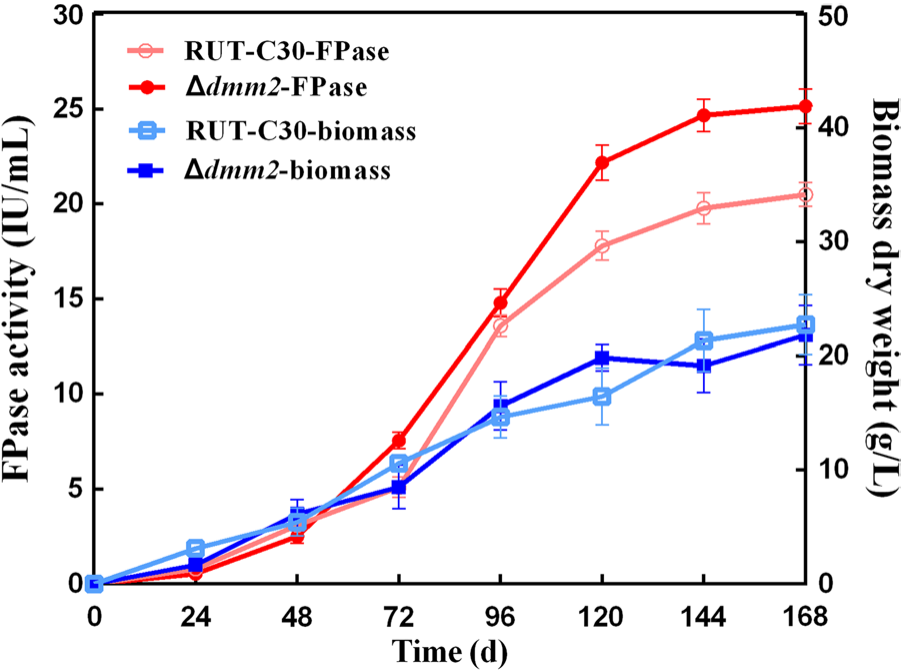
Time-course of the fed-batch culture of *T. reesei* Δ*dmm2* for cellulase production in a 7-L fermenter. The samples were taken at regular intervals, and the supernatant was analyzed for determining the FPase activity. Mycelia were collected for biomass measurement. Values are the mean ± SD of results from three triplicate measurements

To further investigate the potential for industrial applications of Δ*dmm2*, cellulase production was analyzed in a 7**-**L fermenter. Figure 9 illustrates the time course of the fed-batch culture using lactose as the carbon source. For Δ*dmm2*, 15.2 g/L of Δ*dmm2* biomass was obtained at 144 h, and the maximum FPase activity was reached (25.15 U/mL) at 168 h. An increase of approximately 20–30% in cellulase production was observed between Δ*dmm2* and parental strain RUT-C30 (20.51 U/mL) after a 7-day cultivation (Fig. 9). These results indicated that Δ*dmm2* is an effective strain for cellulase production.

## Discussion

In this study, we characterized a three-gene cluster containing *hem8*, *tku70*, and *dmm2*, and investigated the functions of this cluster in *T. reesei*. Transcript-level analysis of these three tandem genes showed that they are all induced by blue light. *tku70*, the ortholog of human KU70 in *T. reesei*, is required for the NHEJ pathway of DNA repair. Thus, we used deletion mutants and complementation experiments to examine the functions of *hem8* and *dmm2*, which were coregulated with *tku70* by blue light.

Using BLAST comparisons, we found that *hem8* encodes a putative ferrochelatase (EC 4.99.1.1). Mutation of this gene causes photosensitivity, such as porphyria in humans, likely due to the accumulation of porphyrin intermediates, that are highly phototoxic [12–14]. We found that *hem8* is under the control of not only a 496-bp light-inducible bidirectional promoter between *hem8* and *tku70*, but also of two crucial LRE motifs overlapped with the ORF of *tku70.* Blue light mediates *hem8* expression, resulting in the reduction of photo-oxidative damage. Furthermore, in 75% (136/180) of ascomycetous fungi, the ortholog genes of *hem8* and *tku70* are in close proximity (no longer than 1000 bp) and are encoded in two different DNA strands. These results illustrate that the overlap between *hemH* and *ku70* may be universal in ascomycetous fungi, and the overlap associated with coregulation may be explained by natural selection.

Deletion of *hem8* was successful in the Δ*tku70* strain and we could obtain homokaryon transformants with 250 mg/L of hematin supplementation, whereas ferrochelatase mutants have been reported to be difficult to achieve in *C. neoformans* [13] and *A. niger* [14]. We demonstrate that complementation of *hem8* could be achieved via different promoters, such as *pdc* and *xyn2* (data not shown), in addition to its own endogenous promoter, used in p3C*hem8*. Researchers were unable to directly isolate ferrochelatase-deletion mutants in haploid and diploid strains of *C. neoformans* through conventional gene replacement experiments using HR [13]. Two reasonable explanations for these problems would be that *C. neoformans ku70* mutation strains were not used as hosts, and that 10 mg/L of hematin supplementation was implemented [13]. In contrast, in our study, we used 250 mg/L of hematin, which was a significantly higher concentration. In *A. niger*, the selection of homokaryons was achieved by increasing the hematin concentration up to 1 g/L and adding 1% (w/v) Tween 80 for 10–21 days of incubation [14]. We could obtain homokaryons of *T. reesei* with a simple deletion of ferrochelatase, when compared to that in *A. niger* [14]. Using medium without added carbohydrates and malt extract agar supplemented with 250 mg/L of hematin, homokaryon transformants were easily obtained after 3–5 days of incubation. In previous studies in *A. niger*, researchers used minimal medium or complete medium with glucose as the carbon source [14]. Thus, additional studies are required to determine whether the isolation would be easy in *A. niger* if a medium without added glucose was used.

Using BLAST comparisons, we identified *tre108087* as a putative DNA methylation modulator-2 gene *dmm2*, which was shown to be widely present in fungi, and it was reported to prevent the spread of DNA methylation from transposons to nearby genes in *N. crassa* [15, 16].

We systematically studied the role of *tku70* and its neighbor, *dmm2*, in DNA repair using UV irradiation, HU, BLM, MMS, EMS, CPT, and MIT. We observed that the *T. reesei* Δ*tku70* strain showed increased sensitivity to BLM, when compared to the parental RUT-C30 strain. Because KU70 is a key component of the NHEJ pathway-associated DSB repair, it was not unexpected that the *T. reesei* Δ*tku70* strain showed increased sensitivity to the DSB inducer BLM. DMM2 plays a positive role in DNA repair. Notably, the *T. reesei* Δ*dmm2* mutant was also sensitive to BLM and showed increased sensitivity to HU and CPT, which are expected to interact physically and/or genetically with DNA synthesis and topoisomerase I. Results of the epistasis analysis indicated that *tku70* and *dmm2* play different roles in DSB repair.

DMM2 plays an inhibitory role in cellulase production and the expression of the cellulase crucial activators *xyr1* and *ace3* in *T. reesei.* The *T. reesei* Δ*dmm2* mutant showed significant improvement in cellulase production, when compared to the parental strain RUT-C30. Further studies are needed to elucidate the function of the DNA methylation modulator DMM2 in cellulase production.

## Conclusions

In conclusion, we identified a light-regulated three-gene cluster including *tku70*, a key component of the NHEJ pathway, and two new genes (*hem8* and *dmm2*), which were found to be related to DNA damage repair in *T. reesei*. *hem8* encodes a putative ferrochelatase with the coding region overlapping with gene *tku70*. The orthologs of HEM8 were described to be involved in relieving phototoxic damage [12–14]. In addition, *hem8* represents an efficient auxotrophic marker for generating transgenic fungi. Contrarily, *dmm2* encodes a putative DNA methylation modulator-2 gene, which is widespread in the ascomycetous fungal genome (Additional file 1). Our data suggest that this protein is involved in DSB repair and cellulase production in *T. reesei*. Our results provide evidence that DNA methylation modulators affect cellulase expression in *T. reesei*. Further studies are required to elucidate the specific role of *dmm2* in the regulation of gene expression.

## Methods

### Strains and culture conditions

*Escherichia coli DH5α* was used as a host strain for recombinant DNA manipulations. *T. reesei* RUT-C30 (ATCC 56765) was used as a host for gene knockout. The *T. reesei* strains constructed in this study are summarized in Table 1. *Agrobacterium tumefaciens* GV3101 was used in an *Agrobacterium*-mediated transformation system [25]. Luria–Bertani (LB) medium was used for *E. coli* and *A. tumefaciens* cultures. Mandels medium [26] containing a 10 g/L carbon source was used for the general fungal culture. Transformants were selected using Mandels medium containing 100 μg mL^−1^ hygromycin B. Malt extract agar (Merck, Darmstadt, Germany) was used for ferrochelatase mutant culture and mutagen sensitivity analysis. The selective medium for *amdS*^+^ transformation contained (g/L): glucose 20, KH_2_PO_4_ 15, MgSO_4_ 0.6, CaCl_2_, 0.6, FeSO_4_·7H_2_O 0.005, MnSO_4_·H_2_O 0.0016, ZnSO_4_·7H_2_O 0.0014, and CoCl_2_ 0.002, with 10 mM acetamide as the sole nitrogen source and 12.5 mM CsCl. The conditions for *the bar*^+^ transformation are reported earlier by us [32]. The pH of the solution was adjusted to 5.5. In all plates, 1.5% agarose was used as the solidifying agent.

**Table 1:** *T. reesei* strains constructed in this study

Strain	Relevant features	Source
*T. reesei* RUT-C30	Parental strain	ATCC
Δ*tku70-*LRE	*T. reesei* RUT-C30 with pΔ*tku70-2*, amds^+^	This study
Δ*tku70*	*T. reesei* RUT-C30 with pΔ*tku70-3*, amds^+^	This study
Δ*hem8*	*T. reesei* Δ*tku70* with pΔ*hem8*, amds^+^ hyg^+^	This study
Δ*dmm2*	*T. reesei* RUT-C30 with pΔ*dmm2*, hyg^+^	This study
Δ*tku70*&*dmm2*	Δ*tku70* with pΔ*dmm2*, amds^+^ hyg^+^	This study
Δ*hem8*::*1Chem8*	Δ*hem8* with p1C*hem8*, amds^+^ hyg^+^ bar^+^	This study
Δ*hem8*::*3Chem8*	Δ*hem8* with p3C*hem8*, amds^+^ hyg^+^ bar^+^	This study
Δ*dmm2*::*Cdmm2*	Δ*dmm2* with pC*dmm2*, hyg^+^ bar^+^	This study

### Identification and sequence analysis of the *T. reesei* genes

The upstream and downstream sequences of *tku70* in the genome of *T. reesei* (http://genome.jgi.doe.gov/Trire2/Trire2.home.html) were identified using the BLASTP search. The conserved residues were analyzed by alignment of amino acid sequences, and a phylogenetic tree was constructed for protein sequences of some related proteins from three diverse phyla of the fungal kingdom using MEGA version 6.0 and the neighbor-joining method with 1000 bootstrap replicates [27, 28].

### Blue light photoinduction

Spore suspensions of the *T. reesei* strains were used for photoinduction assays after inoculation into 20 mL Mandels medium containing 10 g/L glucose with 10^6^ mL^−1^ conidiospores, at 28 °C for 36 h in the dark before light exposure. Three flasks were then subjected to continuous exposure to blue light in a shaking incubator with light-emitting diodes (blue LED: 137 W m^−2^) at 28 °C. Simultaneously, three flasks were placed in the dark. Samples of mycelia were collected at different times (0, 1, and 2 h), dried on filter paper, and used for RNA extraction. Mycelium collection was performed in a dark room with red light for security.

### RNA isolation and quantitative real-time reverse transcription polymerase chain reaction (RT-qPCR)

Approximately 100 mg of *T. reesei* mycelia were harvested. Total RNA was extracted using a FastRNA Pro Red Kit (MPbio, Irvine, CA, USA), according to the manufacturer’s instructions. Reverse transcription was performed with 1000 ng of total RNA, using TransScript All-in-One First-Strand cDNA Synthesis SuperMix for qPCR (TransGen, Beijing, China), according to the manufacturer’s instructions. For RT-qPCR, the TransStart TipTop Green qPCR SuperMix (TransGen) was used with 200 nM of forward and reverse primers (Additional file 2: Table S1) and 1 μL of tenfold diluted cDNA in a final volume of 20 μL. Under different light conditions, the ribosomal protein-encoding gene *rpl6e* was found to be a reliable reference gene for RT-qPCR in *T. reesei* [18, 29]. For gene transcription analysis, SYBR Green assays with the reference genes *rpl6e* [18, 29] and *sar1* [30] were performed as previously described. Thermocycling was performed in an ABI StepOne Plus thermocycler (Applied Biosystems, Foster City, CA, USA).

### Construction of *T. reesei* Δ*tku70*, Δ*hem8*, Δ*dmm2*, and Δ*hem8*&*dmm2* mutants

The deletion cassettes for the deletion of genes *tku70*, *hem8*, and *dmm2* were constructed using a pEASY-Uni Seamless Cloning and Assembly Kit (TransGen). The *T. reesei* Δ*tku70* mutant was constructed with the *amdS*-selectable marker, and Δ*hem8* and Δ*dmm2* strains were obtained using a hygromycin resistance marker. The deletion construct contained the selection marker (from pamdS or pPK1s) [31] flanked by 1–2-kb fragments upstream and downstream from the 5′ and 3′ ends of the open reading frames (ORFs) of *tku70*, *hem8*, and *dmm2*, respectively (Fig. 2, Additional file 2: Fig S5).

All the primers used are listed in Additional file 2: Table S1. The 5′-flanks were first assembled at the *Pac*I/*Xba*I sites of the pPK1s (pamdS for *tku70*) vector [31]. Subsequently, 3′-flanks were cloned into the *Swa*I site of the three resulting plasmids with corresponding 5′-flanks. The deletion vectors (pΔ*tku70*-1, pΔ*tku70*-2, pΔ*tku70*-3, pΔ*hem8*, and pΔ*dmm2*) were transformed into the *T. reesei* RUT-C30 strain using *Agrobacterium*-mediated transformation. For pΔ*tku70*-1, pΔ*tku70*-2, pΔ*tku70*-3, and pΔ*dmm2* transformation, 24 transformants were collected from the appropriate plates [31], and diagnostic PCR was used to verify the successful knockout of the targets [33]. The principles and primers used for diagnostic PCR are listed in Additional file 2: Fig. S5.

### Isolation of the ***T. reesei*** Δ***hem8*** mutant

The *hem8* deletion strains were constructed by transforming the disruption vector pΔ*hem8* into the Δ*tku70* strain and plated on malt extract agar supplemented with 100 mg/L hygromycin. Colonies (heterokaryons) with hygromycin resistance germinated after 3–7 days. The conidia were then collected, diluted, and spread on malt extract agar supplemented with 0–250 mg/L hematin and 0–10 g/L carbon source (glucose or lactose). The obtained homokaryons were analyzed for porphyrin accumulation using autofluorescence detection and verified using diagnostic PCR [33]. The principles and primers used for diagnostic PCR are listed in Additional file 2: Fig. S5.

### Construction of complementation strains

For complementation of Δ*hem8*, 3.2- and 3.6-kb PCR products of *hem8* were obtained using the primer pairs shown in Additional file 2: Table S1. The two PCR products were similar except that the 3.6-kb PCR product contained a 0.4-kb DNA fragment from the nearby gene *tku70* (Fig. 2). For complementation of the deletion strain Δ*dmm2*, the 4.5-kb PCR product of *dmm2* was obtained using specific primer pairs (Additional file 2: Table S1). The two PCR products of *hem8* and one PCR product of *dmm2* were assembled at the *Pac*I/*Xba*I sites of p9B [32] to obtain the complementation vectors p1C*hem8*, p3C*hem8*, and pC*dmm2* (Fig. 2), which were transformed into the corresponding deletion strains of *T. reesei*.

Complementation of the Δ*hem8* strain was obtained using *hem8* as the selection marker and was verified by diagnostic PCR and full restoration of growth on medium without hematin. Complementation of the Δ*dmm2* strain was performed using *a bar* as the selection marker [32] and was verified by diagnostic PCR with primers pair *dmm2-*OF/OR.

### Mutagen sensitivity

The survival rates of the strains in the presence of MMS, EMS, BLM, camptothecin (CPT), mitoxantrone (MIT), and hydroxyurea (HU) were measured as described by Suzuki et al. [8]. The survival rates of different *T. reesei* strains after exposure to UV irradiation were measured as previously described [4]. UV exposure was determined using a UV crosslinker (UVP, Upland, CA, USA). Conidial suspensions were adjusted to an OD_600_ of 0.8 and then diluted tenfold. Aliquots were sampled and plated on malt extract medium. The surviving colonies were counted after 3 days. All survival experiments were performed in triplicate.

### Cellulase production in a shake flask and fermenter culture

Cellulase production in a shake flask was conducted as previously described [2]. In brief, conidia (final concentration 10^6^/mL) of *T. reesei* strains were grown at 28 °C, in 20 mL of 2 × Mandels medium (1.0 g/L yeast extract, 3.0 g/L peptone, 0.6 g/L urea, 2.8 g/L (NH_4_)_2_SO_4_, 4.0 g/L KH2PO4, 0.5 g/L CaCl_2_, 0.6 g/L MgSO_4_·7H_2_O, 5.0 mg/L FeSO_4_·7H2O, 1.6 mg/L MnSO_4_·4H2O, 1.4 mg/L ZnSO_4_·7H_2_O, and 20 mg/L CoCl_2_·6H_2_O) containing 2% (w/v) lactose or 1% (w/v) Avicel (PH-101, Sigma-Aldrich) as the sole carbon source. The supernatant was used for the cellulase assays. Filter paper hydrolyzing activity (FPA) was measured using 1 × 6 cm filter paper (Whatman No. 1; Whatman Laboratories, Hillsboro, OR, USA) in 5-mL reactions with 0.1 M K_2_HPO_4_-KH_2_PO_4_ buffer (pH 5.0) at 50 °C for 60 min. The released reducing sugar was determined by the 3, 5-dinitrosalicylic acid (DNS) colorimetric method using glucose as a standard. One unit of activity (U) was defined as the amount of enzyme that released 1 µmol of glucose-equivalent reducing sugar per minute [2]. Mycelia were collected for RNA extraction.

Cellulase production in a fermenter culture was conducted according to the method described in a previous study [2] with some modifications. In brief, fermentation was conducted in a 7-L fermenter (Shanghai Bailun Bio-technology Co., Ltd.) with an initial working volume of 3 L at 28 °C for mycelial growth. Seed cultivation was performed as follows: for each strain, approximately 10^9^ conidia were inoculated into 300 mL Mandels medium with 10 g/L glucose and 10 g/L lactose and then cultivated using rotation (200 rpm) at 28 °C for 36 h. This culture was poured into 2.7 L of fresh 2 × Mandels medium containing 5 g/L glucose, 37 g/L lactose, and 27 g/L corn steep in a 7-L jar fermenter and cultivated using rotation at 28 °C, 200–500 rpm, and 1 vvm for 7 days. Feeding took place after 3 days of fermentation by adding 60 g lactose and 10 g corn syrup every 24 h. The pH was controlled within the range of 4.0–4.3 for the first 3 days and at 5.0–5.2 thereafter with ammonia water. The dissolved oxygen (DO) amount was controlled above 10%. The supernatant was used for the cellulase assay and protein concentration determination. Subsequently, mycelia were collected for biomass measurements.

### Statistical analysis

All experimental data shown in this paper were obtained from at least three independent samples with identical or similar results. The error bars indicate standard deviations (SDs) from the mean of triplicate determinations. Student’s *t* test was used to compare two samples. Duncan’s multiple-range test was used for multiple comparisons. Within each set of experiments, *P* < 0.05 was considered to indicate a significant difference.

## Supplementary Information

**Additional file 1:** Genome-wide surveys of the homologues of the three-gene cluster in 324 sequenced fungal genomes.

**Additional file 2: Figure S1.** **A** *T.* *reesei* *hem8* is essential and Δ*hem8* mutants exhibit an extreme growth defect in the absence of haematin. **B** Heterokaryons showed slow growth with supplementation of haematin. **C** Heterokaryons displayed slight red auto-fluorescence under 365 nm UV light. **D** Spores were inoculated on malt extract agar plates with 250 mg L^−1^ and homokaryons were isolated. Colonies displayed obvious red auto-fluorescence under 365 nm UV light. **Figure S2.** Cassette p3C*hem8* and p1C*hem8* complemented the Δ*hem8* mutant. **A** The complementation vectors (p3C*hem8* and p1C*hem8*) were transformed to *T. reesei* Δ*hem8* mutant using *Agrobacterium*-mediated transformation using *hem8* as the selection marker. **B** Conidia (5 × 10^3^) of strains with cassette p3C*hem8* (left) or p1C*hem8* (right) were spread on Mandels’ medium plates without hematin. **C** Red auto-fluorescence was detected under 365 nm UV light for two kinds of complementation strains. **Figure S3.** Sensitivities and epistasis analysis of Δ*tku70* and Δ*dmm2*. A conidial suspension was mixed with malt extract agar medium containing the colony restrictor Triton X-100 and MMS, EMS or MIT at indicated concentration. Colonies were counted after incubation at 28 °C for 2–3 days. All error bars indicate mean ± SEM (*n* = 3 samples) from the same experiment. **Figure S4.** The biomass production of *T. reesei* RUT-C30 and Δ*dmm2 *with lactose as the carbon source. Mycelia were collected for biomass measurement. Values are the mean ± SD of results from three triplicate measurements. **Figure S5.** Diagnostic PCR for *tku70*, *hem8*, and *dmm2* deletion. The gene deletion cassettes for *tku70*, *hem8*, and *dmm2* were constructed by ligating approximately 1000 bp of the 5′- and 3′-flanks into the backbone plasmids (pPK1s for *hem8* and pamdS for *tku70*). The binding sites of primers on the genome of *T. reesei* and the expected sizes of the products in diagnostic PCR for gene deletions are shown. Diagnostic PCR for *tku70 *deletion was conducted using the following primer pairs: *tku70*-CF/D71 for the region upstream of the 5′-end, HG3.5/*tku70*-CR for the region downstream of the 3′-end, and *tku70*-OF/OR for the open reading frame of *tku70*. Diagnostic PCR for *hem8 *deletion was conducted using the following primer pairs: *hem8*-CF/D72 for the region upstream of the 5′-end, HG3.6/*hem8*-CR for the region downstream of the 3′-end, and *hem8*-OF/OR for the open reading frame of *hem8*. Diagnostic PCR for *dmm2 *deletion was conducted using the following primer pairs: *dmm2*-CF/D72 for the upstream region of the 5′-end, HG3.6/*dmm2*-CR for the downstream region of the 3′-end, and *dmm2*-OF/OR for the open reading frame of *dmm2*. **Table S1.** Primers used in this study.

## Data Availability

All data generated or analyzed during this study are included in this published article (and its Additional files).

## Abbreviations

UV: Ultraviolet
FPA: Filter paper activity
MA: Mandels–Andreotti
qPCR: Quantitative PCR
ORF: Open reading frame
BLASTP: Protein basic local alignment search tool
