# Supplementary material for: A three-gene cluster in Trichoderma reesei reveals a potential role of dmm2 in DNA repair and cellulase production
Source: Biotechnol Biofuels Bioprod. 2022 Mar 29;15:34. doi: 10.1186/s13068-022-02132-y (PMC8966179; doi:10.1186/s13068-022-02132-y)
Supplement: Supplementary file 2 — Additional file 2: Figure S1. A T. reesei hem8 is essential and Δhem8 mutants exhibit an extreme growth defect in the absence of haematin. B Heterokaryons showed slow growth with supplementation of haematin. C Heterokaryons displayed slight red auto-fluorescence under 365 nm UV light. D Spores were inoculated on malt extract agar plates with 250 mg L−1 and homokaryons were isolated. Colonies displayed obvious red auto-fluorescence under 365 nm UV light. Figure S2. Cassette p3Chem8 and p1Chem8 complemented the Δhem8 mutant. A The complementation vectors (p3Chem8 and p1Chem8) were transformed to T. reesei Δhem8 mutant using Agrobacterium-mediated transformation using hem8 as the selection marker. B Conidia (5 × 10^3^) of strains with cassette p3Chem8 (left) or p1Chem8 (right) were spread on Mandels’ medium plates without hematin. C Red auto-fluorescence was detected under 365 nm UV light for two kinds of complementation strains. Figure S3. Sensitivities and epistasis analysis of Δtku70 and Δdmm2. A conidial suspension was mixed with malt extract agar medium containing the colony restrictor Triton X-100 and MMS, EMS or MIT at indicated concentration. Colonies were counted after incubation at 28 °C for 2–3 days. All error bars indicate mean ± SEM (n = 3 samples) from the same experiment. Figure S4. The biomass production of T. reesei RUT-C30 and Δdmm2 with lactose as the carbon source. Mycelia were collected for biomass measurement. Values are the mean ± SD of results from three triplicate measurements. Figure S5. Diagnostic PCR for tku70, hem8, and dmm2 deletion. The gene deletion cassettes for tku70, hem8, and dmm2 were constructed by ligating approximately 1000 bp of the 5′- and 3′-flanks into the backbone plasmids (pPK1s for hem8 and pamdS for tku70). The binding sites of primers on the genome of T. reesei and the expected sizes of the products in diagnostic PCR for gene deletions are shown. Diagnostic PCR for tku70 deletion was conducted using the following primer pa [file 13068_2022_2132_MOESM2_ESM.docx]

**A three-gene cluster in *Trichoderma reesei* reveals a potential role of *dmm2* in DNA repair and cellulase production**

**Wanchuan Cai, Yumeng Chen, Lei Zhang, Xu Fang, Wei Wang***

*State Key Lab of Bioreactor Engineering, New World Institute of Biotechnology,* *East China University of Science and Technology, Shanghai 200237, China*

*****Corresponding author: Wei Wang

Mailing address: East China University of Science and Technology, P.O. Box 311, 130 Meilong Road, Shanghai 200237, China

Phone: +86-21-64251923, Fax: +86-21-64250068.

E-mail: wadexp@ecust.edu.cn

1. **Table S1. Primers used in this study.**
2. **Figure S1.** (A) *T. reesei* *hem8* is essential and Δ*hem8* mutants exhibit an extreme growth defect in the absence of haematin. (B) Heterokaryons showed slow growth with supplementation of haematin. (C) Heterokaryons displayed slight red auto-fluorescence under 365 nm UV light. (D) Spores were inoculated on malt extract agar plates with 250 mg l^−1^ and homokaryons were isolated. Colonies displayed obvious red auto-fluorescence under 365 nm UV light.
3. **Figure S2. Cassette p3C*hem8* and p1C*hem8* complemented the Δ*hem8* mutant.** (A) The complementation vectors (p3C*hem8* and p1C*hem8*) were transformed to *T. reesei* Δ*hem8* mutant using *Agrobacterium*-mediated transformation using *hem8* as the selection marker. (B) Conidia (5×10^3^) of strains with cassette p3C*hem8* (left) or p1C*hem8* (right) were spread on Mandels’ medium plates without hematin. (C) Red auto-fluorescence was detected under 365 nm UV light for two kinds of complementation strains.
4. **Figure S3. Sensitivities and epistasis analysis of Δ*tku70* and Δ*dmm2*.** A conidial suspension was mixed with malt extract agar medium containing the colony restrictor Triton X-100 and MMS, EMS or MIT at indicated concentration. Colonies were counted after incubation at 28°C for 2 to 3 days. All error bars indicate mean ± SEM (n = 3 samples) from the same experiment.
5. **Fig. S4 The biomass production of *T. reesei* RUT-C30 and Δ*dmm2* with lactose as the carbon source.** Mycelia were collected for biomass measurement. Values are the mean ± SD of results from three triplicate measurements.
6. **Fig. S5 Diagnostic PCR for *tku70*, *hem8*, and *dmm2* deletion.** The gene deletion cassettes for *tku70*, *hem8*, and *dmm2* were constructed by ligating approximately 1000 bp of the 5′- and 3′-flanks into the backbone plasmids (pPK1s for *hem8* and pamdS for *tku70*). The binding sites of primers on the genome of *T. reesei* and the expected sizes of the products in diagnostic PCR for gene deletions are shown. Diagnostic PCR for *tku70* deletion was conducted using the following primer pairs: *tku70*-CF/D71 for the region upstream of the 5′-end, HG3.5/*tku70*-CR for the region downstream of the 3′-end, and *tku70*-OF/OR for the open reading frame of *tku70*. Diagnostic PCR for *hem8* deletion was conducted using the following primer pairs: *hem8*-CF/D72 for the region upstream of the 5′-end, HG3.6/*hem8*-CR for the region downstream of the 3′-end, and *hem8*-OF/OR for the open reading frame of *hem8*. Diagnostic PCR for *dmm2* deletion was conducted using the following primer pairs: *dmm2*-CF/D72 for the upstream region of the 5′-end, HG3.6/*dmm2*-CR for the downstream region of the 3′-end, and *dmm2*-OF/OR for the open reading frame of *dmm2*.

**Table S1. Primers used in this study.**

| Name |  | Sequences (5’-3’) |  | Relevant features |
| --- | --- | --- | --- | --- |
| 5-*tku70*-1f |  | GATTACGAATTCTTAATTAATCGCTCCATGACGGCTTGAAC |  | 5’ –flanks for *tku70* deletion |
| 5-*tku70*-1r |  | TTAAGTTAACTCTAGATATGTCTGATTTGGGTTTCAGC |  |  |
| 5-*tku70*-2f |  | GATTACGAATTCTTAATTAACTTTTCCGTAACCGTCGTCTCAGG |  |  |
| 5-*tku70*-2r |  | TTAAGTTAACTCTAGATCCTGCTCTGCTTCATCGTCCT |  |  |
| 5-*tku70*-3f |  | GATTACGAATTCTTAATTAAATATATTACAGATGCCATGCTCTC |  |  |
| 5-*tku70*-3r |  | TTAAGTTAACTCTAGAACTGGCGGAGGCTCAAGCAT |  |  |
| 3-*tku70*-f |  | ACTAGTGAGCTCATTTCTTGGTGCGTGCTTCGATCTAAC |  | 3’ –flanks for *tku70* deletion |
| 3-*tku70*-r |  | AGTGCCAAGCTTATTTCCTCTGTGAATACTCGGCAACCA |  |  |
| *tku70*-CF |  | GCCACCCAGCCCTATTCACAAG |  | Diagnostic PCR for *tku70* deletion |
| D71 |  | CTGAAGCAACAGGTGCCAAGGA |  |  |
| HG3.5 |  | AGTTGCTGGGAAATGTGGTGACTC |  |  |
| *tku70-*CR |  | GGCAGCATCCTTCTTCTTCTTCCT |  |  |
| *tku70-*OF |  | GGACGAAGACGAAGACGAAGTG |  |  |
| *tku70-*OR |  | AGATGGAACAATGCTCGCTTGG |  |  |
| 5-*hem8*-f |  | GATTACGAATTCTTAATTAACACTTCGTCTTCGTCTTCGTCCTC |  | 5’ –flanks for *hem8* deletion |
| 5-*hem8*-r |  | TTAAGTTAACTCTAGACGCTGAGAAGAGCAGAATGGCATT |  |  |
| 3-*hem8*-f |  | ACTAGTGAGCTCATTTGGCACCGTGTCGGATATTAGC |  | 3’ –flanks for *hem8* deletion |
| 3-*hem8*-r |  | AGTGCCAAGCTTATTTTAGCAGATAACGACGCGTGTC |  |  |
| *hem8*-CF |  | TTTGGCTCTCAGGGCACATGG |  | Diagnostic PCR for *hem8* deletion |
| D72 |  | TGGCTTCACATTCTCCTTCGCTTA |  |  |
| HG3.6 |  | TGCCTAGTGAATGCTCCGTAACA |  |  |
| *hem8*-CR |  | GCCGCCTTTGTGGTGAATATCT |  |  |
| *hem8*-OF |  | TTCCGTAACCGTCGTCTCAGG |  |  |
| *hem8*-OR |  | GCAACCGTTCAAGCCGTCAT |  |  |
| 5-*dmm2*-f |  | GATTACGAATTCTTAATTAACTGAGATGACGGGTTCTGATGTTGT |  | 5’ –flanks for *dmm2* deletion |
| 5-*dmm2*-r |  | TTAAGTTAACTCTAGACACGAGCTGGTTGGCAATGGAT |  |  |
| 3-*dmm2*-f |  | ACTAGTGAGCTCATTTAACGCACAGGTTCGCAGACG |  | 3’ –flanks for *dmm2* deletion |
| 3-*dmm2*-r |  | AGTGCCAAGCTTATTTCGACGCATACTCACGGCTTCAT |  |  |
| *dmm2*-CF |  | CCCGCAAAGCAGAGAAAGGTAGC |  | Diagnostic PCR for *dmm2* deletion |
| D72 |  | TGGCTTCACATTCTCCTTCGCTTA |  |  |
| HG3.6 |  | TGCCTAGTGAATGCTCCGTAACA |  |  |
| *dmm2*-CR |  | TGCCAGGTTGAGGAAGTTGAGTA |  |  |
| *dmm2*-OF |  | GCAGCAGTTGTAGCAGCAGTC |  |  |
| *dmm2*-OR |  | CCGTCGTTGTCGTCGTTGTC |  |  |
| C*hem8*-f |  | GATTACGAATTCTTAATTAATAATAATAAGACGAAGGGGGCAGG |  | for p1C*hem8* and p3C*hem8* |
| 1C*hem8*-r |  | TTAAGTTAACTCTAGATCAGCACTTCGTCTTCGTCTTCG |  |  |
| 3C*hem8*-r |  | TTAAGTTAACTCTAGAGAGACTGGCGGAGGCTCAAG |  |  |
| C*dmm2*-f |  | GATTACGAATTCTTAATTAAGCGGATAGAGCAGTGGGTTGAG |  | for pC*dmm2* |
| C*dmm2*-r |  | TTAAGTTAACTCTAGAAGCATTATCGTTCCTACCCAT |  |  |
| q-*tku70*-f |  | ATCCAGGTCCTTGAAGCGAGAG |  | RT-qPCR for the three-gene cluster |
| q-*tku70*-r |  | GCTGTGCCACTGTCATCTTCG |  |  |
| q-*hem8*-f |  | GGATACGGGTCGCCTCTGTT |  |  |
| q-*hem8*-r |  | CACCATTACGTGGAGTGTCATTGA |  |  |
| q-*dmm2*-f |  | GAGTCAAGTGCCCGAGGGAAT |  |  |
| q-*dmm2*-r |  | CCTGCTCGTCCTCTGTTGTGA |  |  |
| q-*rpl6e*-f |  | GATACGTCATCGCCACCTCC |  |  |
| q-*rpl6e*-r |  | CTTCTCCTTGGCCTTCTCG |  |  |
| q-*cbh1*-f |  | CTCCATCTCCGAGGCTCTTACC |  | RT-qPCR for the cellulases |
| q-*cbh1*-r |  | GCAAGTGCCGCCATATCTGTTAT |  |  |
| q-*cbh2*-f |  | GCATATTACGCCTCTGAAGTTAGCA |  |  |
| q-*cbh2*-r |  | GCATAGTTACCGCCATTCTTGTTG |  |  |
| q-*egl1*-f |  | GCAGCCTCACCATGAACCAGTA |  |  |
| q-*egl1*-r |  | CACCGTCAGAGTCCAGGAGATAC |  |  |
| q-*egl2*-f |  | TGAACAAGTCCGTGGCTCCAT |  |  |
| q-*egl2*-r |  | ACAATTCGTAGGTCCGCTCCAA |  |  |
| q-*xyr1*-f |  | CTTCCTCCTCCTGCTCATCG |  |  |
| q-*xyr1*-r |  | TCGTGTGCCCTAACAATGGTC |  |  |
| q-*ace3*-f |  | GCCAAGTGCGAGTACCTCAG |  |  |
| q-*ace3*-r |  | GCTGGTCGCTCTTCTTCCTC |  |  |
| q-*sar1*-f |  | TGGATCGTCAACTGGTTCTACGA |  |  |
| q-*sar1*-r |  | GCATGTGTAGCAACGTGGTCTTT |  |  |

**
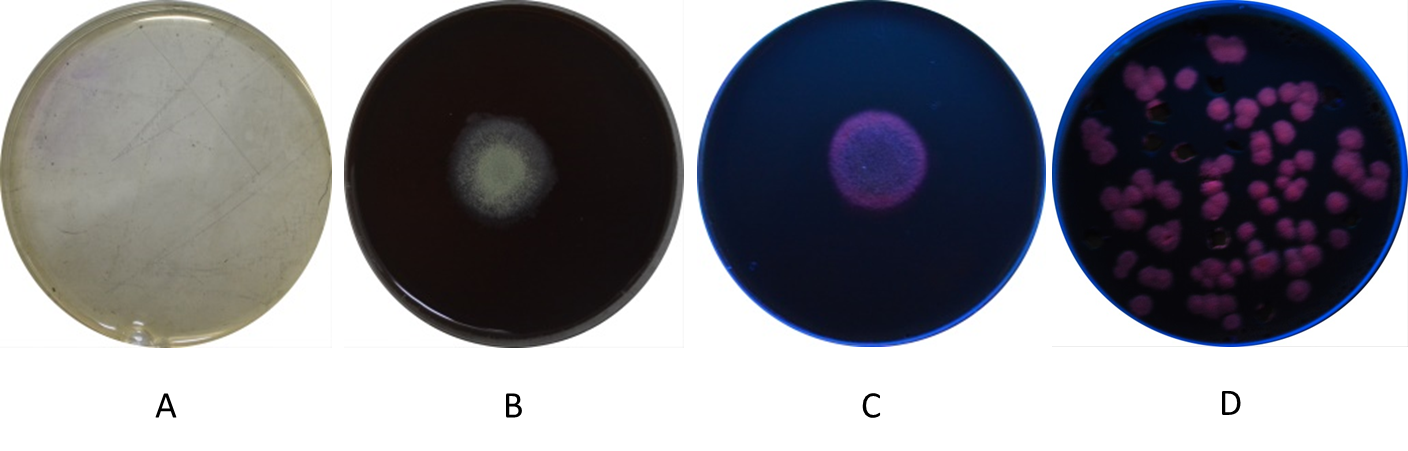
**

**Fig. S1**

**
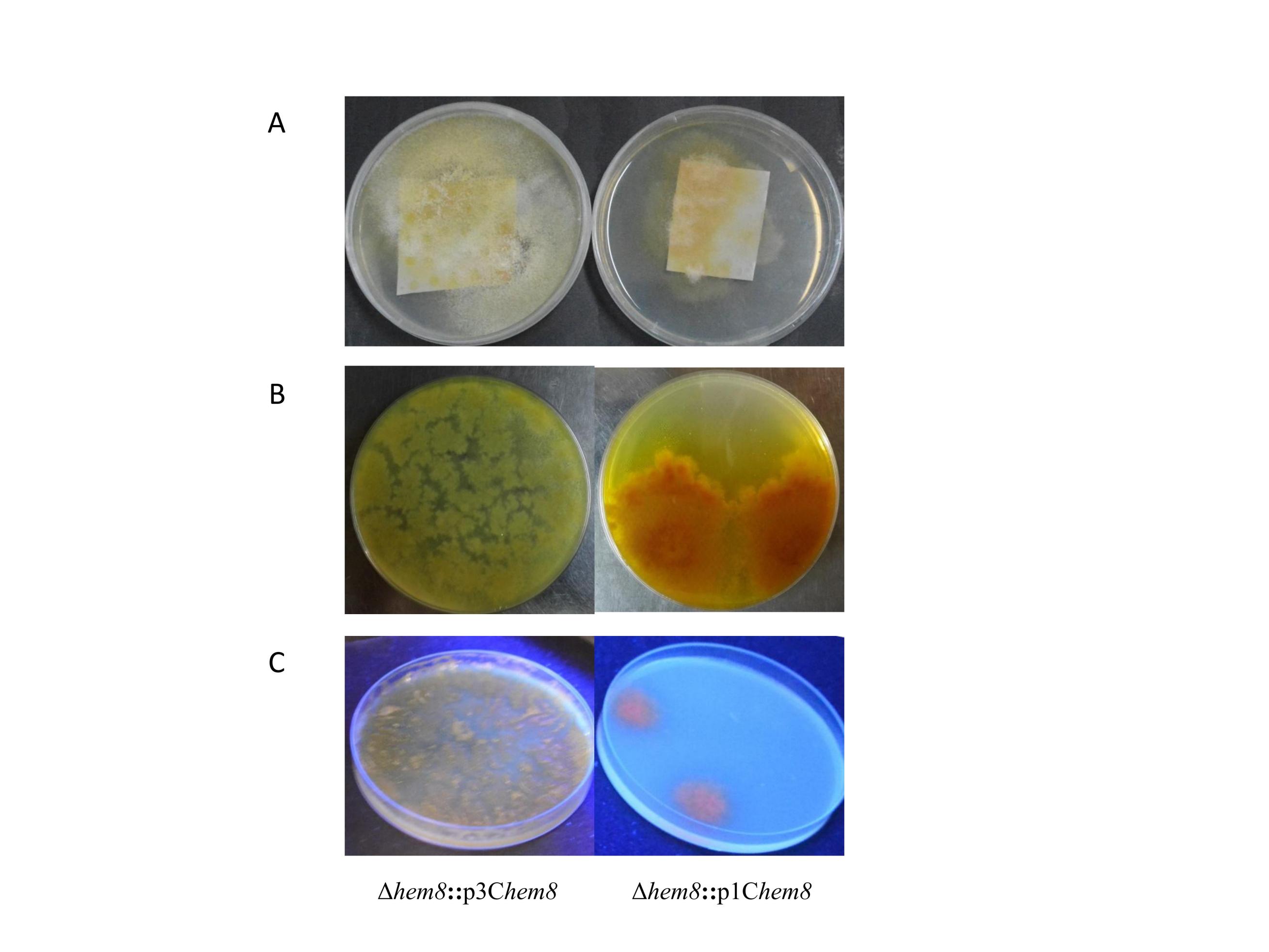
**

**Fig. S2**


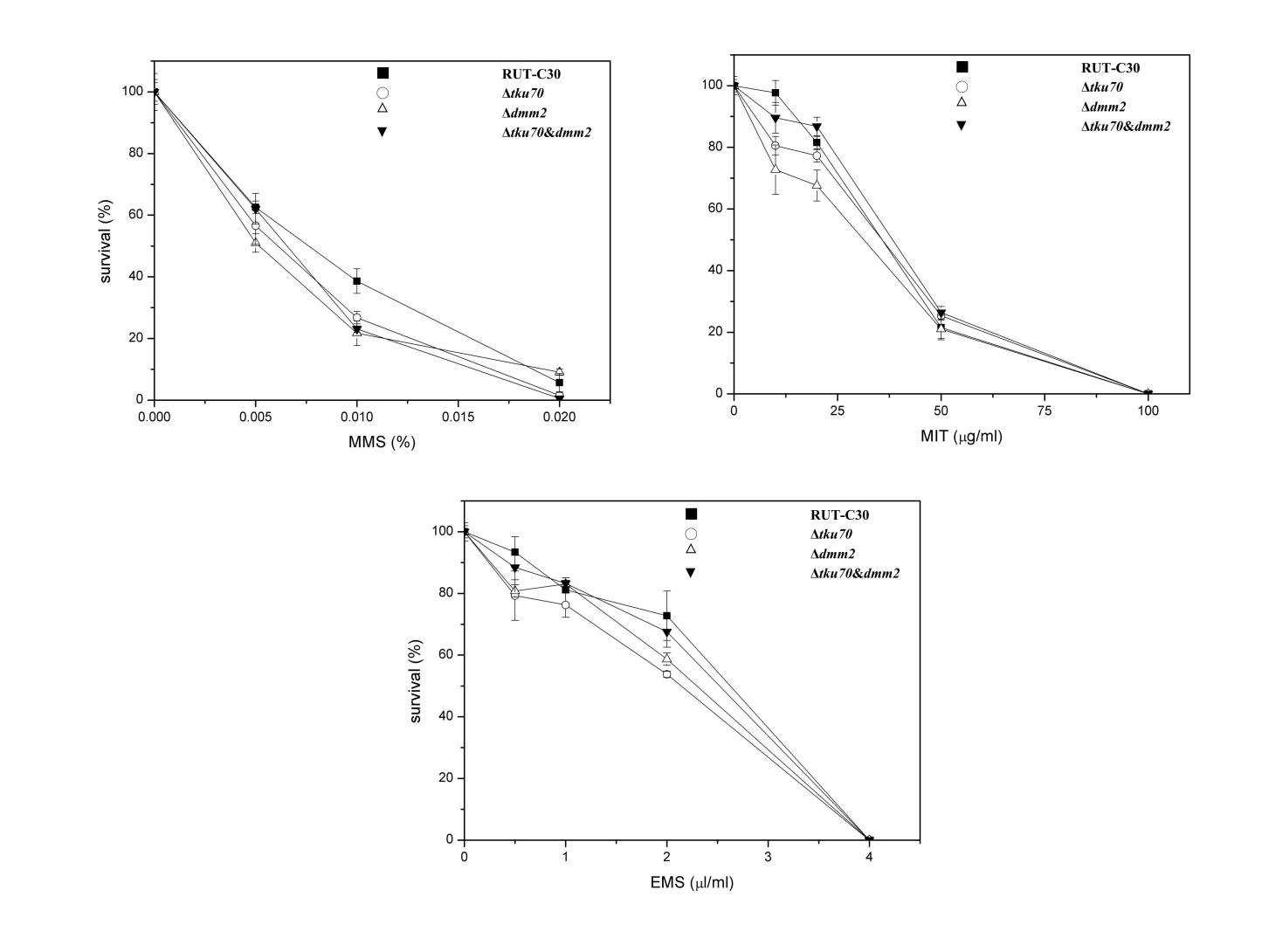


**Fig. S3**





**Fig. S4**


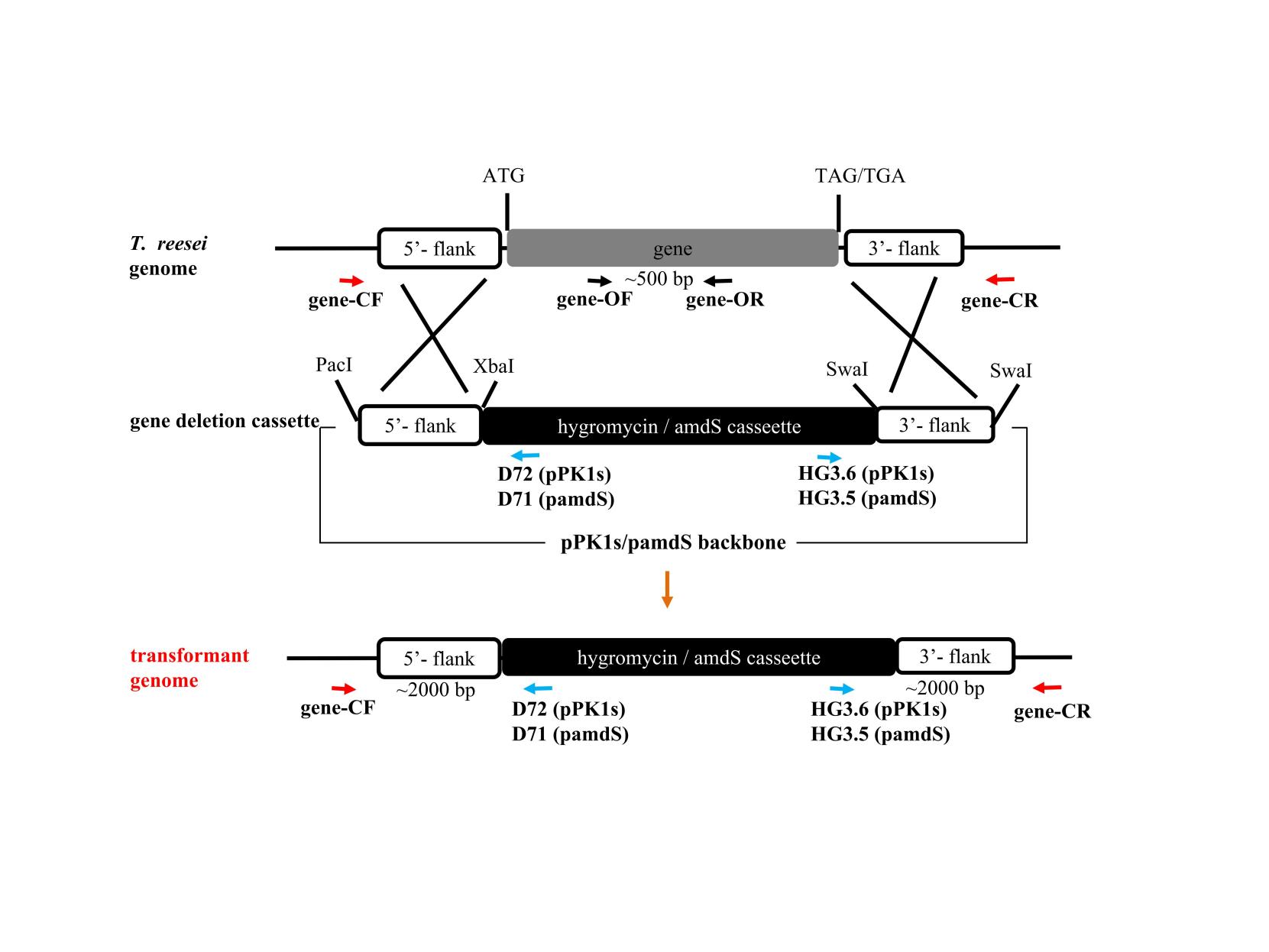


**Fig. S5**
